# Alcohol Withdrawal Presenting with Cut Throat Injury during COVID-19 Lockdown: Case Reports from Nepal

**DOI:** 10.1155/2023/5514321

**Published:** 2023-10-27

**Authors:** Dipesh Bhattarai, Shreeram Upadhyaya, Hemanta Banstola, Sagun Ballav Pant

**Affiliations:** ^1^Department of Psychiatry, Tribhuvan University Teaching Hospital, Kathmandu, Nepal; ^2^Department of General Practice and Emergency Medicine, Tribhuvan University Teaching Hospital, Kathmandu, Nepal

## Abstract

**Background:**

The relationship between alcohol dependence and suicidal tendency is well recognized. Self-harm by cut throat is an uncommon but is potentially life-threatening when attempted. We present a description and discussion of a series of three cases of alcohol dependence syndrome who presented with self-inflicted cut throat wounds during the lockdown period from 24^th^ March to 7^th^ July 2020 due to the COVID-19 pandemic at the largest tertiary care hospital in Nepal. *Case description*. During the three and a half months of COVID-19 lockdown, we had three cases of alcohol dependence syndrome presenting to emergency services with a self-inflicted cut throat injury. Two cases were diagnosed as having alcohol withdrawal delirium and one case as alcohol-induced psychotic disorder (alcoholic hallucinosis) as per the international classification of mental and behavioral disorders diagnostic criteria for research. All three cases were alcohol dependent for more than a decade, but with no prior self-harm attempts. Necessary surgical interventions were done by the Department of Otorhinolaryngology, and in liaison with the Department of Psychiatry, appropriate psychiatric management was done. All three cases had uneventful outcomes in regard to wound care and mental disorder.

**Conclusion:**

Suicidal precautions should be taken in alcohol dependence during phases of consumption and abstinence. Screening for alcohol dependence and withdrawal should be a standard process in all self-harm cases that present to the emergency department during a crisis.

## 1. Introduction

Many nations implemented lockdowns and physical separation during the 2019 coronavirus (COVID-19) pandemic [1]. On January 23, 2020, Nepal became the first nation in South Asia to announce a confirmed case of COVID-19. On March 24, the first nationwide lockdown started. The administration believed that this drastic measure of going into lockdown was essential to increasing the nation's ability to respond to the pandemic [2]. This COVID-19 transmission mitigation approach brought up a critical situation for those who experience substance dependence [3]. Some high-income nations implemented harm reduction programs through modifications to policy and service delivery, while others embraced on forced abstinence [4].

The consumption of alcohol in Nepal is very common, with one in eight men regularly taking alcohol. The bulk of drinkers consume unreported alcohol, which is made up of freely accessible homebrewed beverages from local stores [5]. The prevalence of alcohol dependence was 4.2% among adults, underlining it as a public health issue in Nepal [6]. Following pandemic-associated alcohol prohibition and restriction, it was likely that many individuals with alcohol dependence would be at risk of developing alcohol withdrawal syndrome. At the same time, access to health services was limited due to the lockdown measures [1]. The COVID-19 pandemic was recognized as having seriously jeopardized alcohol dependence treatment and recovery [3, 8]. Notwithstanding, many nations failed to issue policies for the treatment of alcohol withdrawal symptoms during COVID-19 limitations and lockdown periods [1]. Therefore, managing cases of substance dependence during the pandemic and lockdown was extremely difficult in low-resource countries like Nepal [4].

Overall, suicide with sharp-force injuries is uncommon. Emerging evidence showed that 2%–3% of all suicidal cases involved sharp force injuries [9]. Cut throat injuries are potentially fatal due to the several vital structures in the neck [10]. The majority of cut throat cases reported in the prior studies are homicidal rather than suicidal [11, 12]. According to studies, self-harm and suicide have increased during lockdowns by 44% and 71.9%, respectively, demonstrating the worsening mental health situation [13]. The increased risk of suicide during the pandemic has been linked to a number of risk factors, including fear of COVID-19 infection, financial crisis, loneliness, social boycott, quarantine strain, work-related stress, inability to return home due to lockdown, and the lack of alcohol availability and its withdrawal [14]. Alcohol use and dependence dramatically raise the chance of suicidal thoughts, suicide attempts, and suicide [15]. Individuals inflicting stabbing wounds in the abdomen and mutilating their genitals have all occurred during alcohol withdrawal. Alcoholic hallucinosis is typically in the auditory modality and is considered to be harmless. When the hallucination is commanding and threatening, patients may resort to self-harm [16–18]. Thus, in addition to COVID-19, the management of alcohol dependence, with an emphasis on withdrawal, is an equal and immediate concern [19]. Not addressing this issue properly can lead to dire consequences, like suicide. We present here a case series of three patients who presented to our emergency department with a self-inflicted cut throat injury during alcohol withdrawal at the time of the COVID-19 lockdown.

## 2. Cases Descriptions

### 2.1. Case 1

**Figure 1:**
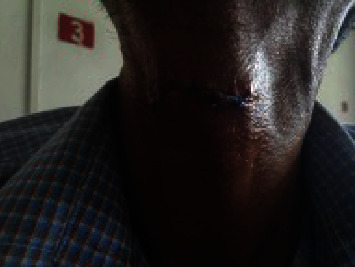
Horizontal laceration over the anterior neck (sutured and closed).

A 50-year-old male from Nuwakot (nearby Kathmandu), married and unemployed, presented with a history of regular alcohol usage, meeting the ICD-10 diagnostic criteria for alcohol dependence over the last 20 years. The Alcohol Use Disorder Identification Test (AUDIT) score of the patient was 30, indicating severe alcohol dependence. He had delirium tremens twice in the last 4 years. In the past, abstinence was prompted by a cultural factor (mourning of a brother) and a physical factor (pancreatitis). This time, the reason for abstinence was the COVID-19 lockdown. Following the lockdown, the patient was forced to stop alcohol due to its unavailability and inaccessibility. Due to movement constraints, family members had trouble getting treatment during the early withdrawal period. After three days of abstinence, the patient started having symptoms of agitation, decreased sleep, tremors of the hand and body, decreased awareness of the surroundings, visual hallucinations, and was hearing frightening voices. While having these symptoms, the patient tried to jump from the house (but was stopped by family members) and later inflicted a cut injury over the neck with a kitchen knife, stating that it was better to die on own than to die from others. A 3^∗^1 cm horizontal laceration in the anterior neck was sustained (Figure 1).

He was rushed to Tribhuvan University Teaching Hospital (TUTH) for an emergency. He was attended by the emergency, otorhinolaryngology, and psychiatry teams. The mental status examination (MSE) at admission showed that the patient had psychomotor agitation, sweating, tremors of both hands and body, disorientation to time and place, impaired attention and concentration, impaired immediate recall, and recent memory. The Clinical Institute Withdrawal Assessment Alcohol Scale Revised (CIWA-AR) score was 45, indicating severe withdrawal. The initial investigations revealed thrombocytopenia (9 lac/cu.mm), alcoholic hepatitis (total bilirubin—1.4 mg/dL, direct bilirubin—0.6 mg/dL, SGOT—269 IU/L, SGPT—56 IU/L), hyponatremia (123 mEq/L), computed tomography (CT) scan of the brain showed cerebral and cerebellar atrophy, and USG abdomen and pelvis showed moderate fatty liver. However, the chest X-ray, other electrolytes, random sugar, serology markers, urine routine examination, electrocardiogram, and thyroid profile were all within the normal range. The COVID-19 PCR was negative. The blood sodium level was normalized on the next day of admission. A provisional diagnosis of alcohol withdrawal delirium was made based on the findings. Treatment was done with intravenous fluids, including 3% normal saline, injectable thiamine 300 mg, intravenous (IV) diazepam 10 mg, injectable haloperidol 5 mg for agitation, injectable ketorolac, wound dressing and suturing under local anesthesia, IV antibiotics, and detoxification with tablet lorazepam 4 mg QID following admission to the psychiatric ward.

Several differential diagnoses were considered: delirium due to hyponatremia, Wernicke encephalopathy, dementia, major depressive disorder, and substance-induced depressive disorder. However, based on the findings that, despite the correction of hyponatremia, delirium persisted, the patient had hyperactive delirium instead of hypoactive delirium, prophylactic injectable thiamine was started in the emergency itself, no previous history of memory impairment or cognitive decline, and the patient's informant initially and the patient (after resolution of delirium) confirmed that he was not depressed. Thus, on the basis of the clinical and laboratory findings the final diagnosis of ICD-10: F10.40 (alcohol dependence syndrome; withdrawal state with delirium without convulsions) was made. The patient recovered well but suffered amnesia regarding the self-harm incident. On the fourth day of admission, symptoms of delirium significantly improved, with no more agitation or hallucinations. Motivational interviewing and naltrexone 50 mg/day were started. The patient was discharged on the 14th day of admission after suture removal and with regular follow-up.

### 2.2. Case 2

**Figure 2:**
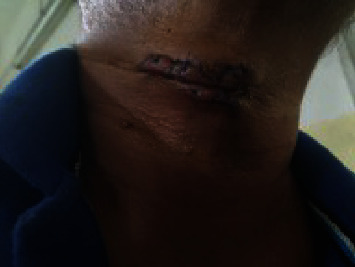
Horizontal lacerations over anterolateral neck (sutured and closed).

A 40-year-old male, separated, non-skilled worker from Dhading presented with a history of alcohol consumption for around 20 years and met the criteria for alcohol dependence syndrome for the last 10 years. His AUDIT score was 24, indicating moderate to severe alcohol dependence. The patient's usual work was disrupted as a result of COVID-19, causing his alcohol relapse. The patient drank alcohol from local neighborhood stores. As a result of the COVID-19 lockdown, shop owners stopped preparing and selling alcohol entirely, resulting in forced abstinence. Following two days of abstinence, he began to claim that the 10–15 individuals were after him and were planning to hurt him. In full consciousness, the patient could hear these voices. The patient fled to hide at a relative's residence nearby. Later, the patient inflicted a cut injury with a kitchen knife, claiming that his act would hold those who followed him accountable for his death. Unlike in the previous case, symptoms arose while the patient was conscious and able to recall all of the events. A 4^∗^1 cm cut in the anteriolateral neck was sustained (Figure 2).

At the time of admission, the MSE showed that the patient was restless, had tremors of the hands and body, was sweating, was oriented to time, place, and person, his attention and concentration were intact, and his CIWA-AR score was 35, indicating severe withdrawal. The initial investigations revealed alcoholic hepatitis (total bilirubin—1.8 mg/dL, SGOT—356 IU/L, SGPT—231 IU/L), Hypokalemia (3.3 mEq/L), USG abdomen and pelvis showed-moderate fatty liver. However, the chest X-ray, other electrolytes, random sugar, serology markers, urine routine examination, electrocardiogram, and thyroid profile were all within the normal range. The CT scan of the brain was normal, and the COVID-19 PCR was negative. Based on the above findings, a provisional diagnosis of alcohol withdrawal syndrome with hallucinosis was made. Treatment was done with intravenous fluids with KCL infusion, injectable thiamine 300 mg, IV diazepam 10 mg, injectable haloperidol 5 mg for agitation, IV ketorolac, wound dressing and suturing under local anesthesia, IV antibiotics, and detoxification was started with a tablet of lorazepam 4-mg QID following admission to the psychiatric ward. The otorhinolaryngology team took care of the wound.

The differential diagnoses considered for this case were delirium tremens, acute and transient psychotic disorder (ATPD), and depressive disorder. However, the intactness of attention, concentration, and other cognitive functions, the patient was not found to be delirious; the development of auditory and visual hallucinations was temporally associated with alcohol abstinence in the background of alcohol dependence, which excluded ATPD, and the patient's informant and patient confirmed that he was not depressed. Thus, on the basis of the clinical findings and laboratory findings, the final diagnosis of F10.56 (Alcohol Dependence Syndrome — Psychotic Disorder: Mixed: hallucination and delusion) was made. On the third day of admission, symptoms of hallucinations significantly improved and subsided. The patient recovered well. The patient was started on baclofen 5 mg twice a day, along with motivational interviewing. Family members were psycho-educated on the risk of severe alcohol withdrawal in the future as a result of unprecedented circumstances such as the COVID-19 lockdown. The patient was discharged on the 14th day of admission after suture removal and with regular follow-up.

### 2.3. Case 3

A 55-year-old male from Trishuli, an uneducated and unskilled worker, presented with a history of using alcohol for about 40 years. An assessment of the patient's history indicated that he had been drinking in a pattern that met ICD-10 criteria for alcohol dependence syndrome for the past 30 years. He had an AUDIT score of 32, indicating severe alcohol dependence.

**Figure 3:**
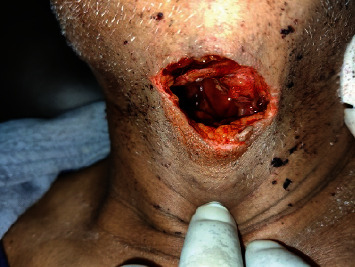
Deep horizontal laceration over the anterior neck exposing the underlying aero vasculature.

As a result of the COVID-19 lockdown, the patient was obliged to reduce his alcohol consumption due to a lack of alcohol at local liquor stores. The patient began suffering withdrawal symptoms but was unable to seek help because of COVID-19 lockout restrictions. After three days, he became disoriented and began experiencing visual and auditory hallucinations. He was hearing terrifying voices telling him that people were coming to kill him. During this time, the patient injured himself on the neck with a *khukuri* (a type of Nepali sharp weapon). A 4^∗^3 cm horizontal laceration in the anterior neck was sustained, exposing the underlying aerovasculature (Figure 3).

The findings of MSE showed the patient was having psychomotor agitation, sweating, tremors of the hand and body, disorientation to time and place, impaired attention and concentration, impaired immediate recall, and recent memory. His CIWA-AR score was 52, indicating that he was experiencing severe alcohol withdrawal. Initial investigations revealed alcoholic hepatitis (SGOT—234 IU/L, SGPT—129 IU/L); USG abdomen and pelvis showed moderate fatty liver. The CT scan of the brain showed cerebral atrophic changes and dilatations of the bilateral ventricles. However, the chest X-ray, electrolytes, random sugar, serology markers, urine routine examination, electrocardiogram, and thyroid profile were all within the normal range, and the COVID-19 PCR was negative. Based on the above findings, a provisional diagnosis of alcohol withdrawal syndrome with delirium was made. Treatment was started with intravenous fluids, injectable thiamine 300 mg, IV diazepam 10 mg, injectable haloperidol 5 mg for agitation, IV ketorolac, wound dressing and suturing under general anesthesia, IV antibiotics, and detoxification was started with tab lorazepam 4-mg QID in a consultant liaison basis. The wound was sutured, and the otorhinolaryngology team performed a tracheostomy. The patient was admitted to the otorhinolaryngology inpatient, and the de-addiction team managed the patient on a liaison basis.

The differential diagnoses considered for this case were delirium due to medical causes, Wernicke encephalopathy, dementia, major depressive disorder, and substance-induced depressive disorder. However, the complete neurological and blood investigations were done to rule out any other cause for delirium. The patient had hyperactive delirium instead of hypoactive delirium, and prophylactic injectable thiamine was started in the emergency. There was no prior history of memory impairment or cognitive decline. The patient's informant initially and patient (after resolution of delirium) confirmed that he was not depressed. Thus, on the basis of the clinical and laboratory findings, the final diagnosis of F10.40 (alcohol dependence syndrome; withdrawal state with delirium without convulsions) was made. Motivational interviewing and the anti-craving agent naltrexone (50 mg/day) were started after the resolution of alcoholic hepatitis.

On the 7^th^ day, symptoms of delirium significantly improved, with no more agitation and hallucinations. Unlike the previous two cases, this case had a deep-cut injury that necessitated a tracheostomy and a longer hospital stay while having the same diagnosis as the first case. The patient was discharged on day twenty of admission with a tracheostomy in situ and with regular follow-up.

## 3. Discussion

As a communal response to the spread of COVID-19 in 2020, Nepal declared a statewide lockdown. The lockdown had a significant impact on people's daily lives in Nepal [2]. Psychiatric patients, in particular, encountered major difficulties, and alcohol dependence management was not adequately acknowledged [3, 4, 8]. All three cases used home-brewed alcohol from local stores. During the lockdown, emergency room visits and hospital admissions for alcohol withdrawal surged [7]. Several factors, including abstention due to fear of acquiring the COVID-19 infection, a shortage of components for manufacturing alcohol at home, could be responsible. Given the state of alcohol consumption in Nepal, the lack of alcohol and forced abstinence may be a major factor in all the cases [5]. This led to severe alcohol withdrawal syndromes and unprecedented consequences, like in our case scenarios.

Another contrasting situation is that some studies have revealed that alcohol consumption surged during the COVID-19 lockdown period, particularly in high-income countries [20–24]. This could be due to variances in alcohol usage, such as homebrewed spirits (Aila/Raksi) being the most common type of alcohol consumed in Nepal [5]. The COVID-19 lockdown affected its availability and accessibility, leading to an unplanned withdrawal. During the lockdown, public transportation was unavailable, creating barriers to accessing health services, and health professionals were overwhelmed. Emergency services were reduced for general health issues and targeted at COVID-19 care, and outpatient services were interrupted [19]. All three patients had complicated withdrawal symptoms and lived outside of the Kathmandu valley. Early intervention may have been impeded by a variety of factors, such as a lack of family education and awareness, geographical difficulty, and a lack of access to health services, but COVID-19 lockdown restrictions in movement may have been the major contributor to the treatment delay, as all cases had previously been admitted to hospitals for complicated withdrawal management. Severe alcohol withdrawal, particularly delirium, poses numerous obstacles, as demonstrated in our cases. During the lockdown, our center did not have in-house testing, and isolation facilities were being built. In addition, our cases involved sharp-force injuries to the neck and needed multidisciplinary intervention. Aside from this, alcohol withdrawal causes vomiting, fever, and tachypnea, all of which are COVID-19 symptoms [25]. Thus, patients who attend the emergency department with COVID-19 symptoms must be screened for alcohol dependence and withdrawal.

It was observed that trauma cases decreased significantly during the COVID-19 lockdown phase [26]. The data on suicide, on the other hand, has been a source of contention. Suicide rates in Nepal soared during the COVID-19 lockdown period. Economic crises and recessions, unemployment, poverty, and other lockdown stressors may be strongly linked to psychological turmoil and suicidal behaviors [27–29]. Another important but neglected factor was substance dependence. Forced and sudden abstinence caused severe withdrawal and psychological pain, driving to suicidal activities like in our cases [14]. Alcohol dependence and problematic alcohol use are well-known risk factors for self-harm and suicide. Alcohol can enhance impulsivity and pain threshold, as well as impair judgment, which can lead to self-harm [15, 30–33]. Nonetheless, in the three cases mentioned, self-harm happened during the withdrawal state. Thus, not only during use and intoxication, but alcohol withdrawal can also be a time when suicide and self-harm can result. Self-mutilation injuries have been recorded in studies during alcohol withdrawal [16–18, 34]. Substance use disorders have been linked with violent methods of self-harm and suicide [16, 34–36]. In all three cases, cut throat is the way of presentation, which is also a violent method of suicide. Two of the cases used kitchen knives, and one used *khukuri*, a local sharp weapon. Both weapon categories are readily available and accessible in the house. Despite this, the most common methods of suicide and self-harm in Nepal are self-poisoning with pesticides, followed by medication overdose and hanging [37]. Alcohol withdrawal hallucinosis, although not regarded as a complicated withdrawal condition, is associated with a significant risk of self-harm if patients experience threatening auditory and visual hallucinations, as in our second scenario, who did not have delirium. All three cases had a psychopathology of threatening auditory hallucinations that led to self-harm. It is well known that this psychopathology increases the likelihood of suicidality [16–18].

All three patients arrived at the emergency room with cut throat injuries. Early screening, identification, and management of substance dependence are critical in all cases of self-harm presenting to the emergency department [38, 39]. Several strategies have been presented to address the issue of substance dependence treatment during a crisis such as COVID-19. Scottish health services distributed information to patients to help them assess their risk of severe alcohol withdrawal and plan safe domiciliary detoxification. [40]. However, not the same approach works for all, and this strategy may only operate in a higher-income, well-educated society [19]. Nepal formally launched its cooperation to execute SAFER, a strategy for the prevention and reduction of alcohol-related harm and mortality [41]. It could be an important step in addressing the public health issue of alcohol dependence in Nepal. Nonetheless, planning, anticipating a surge in severe withdrawal, and ensuring that treatment services are not hindered are all sensible steps to take during crises like the COVID-19 lockdown. In all three cases, family members were given psychoeducation regarding alcohol withdrawal and the problems that can occur in future crisis situations, as well as the importance of seeking timely care. A relapse prevention approach was also implemented in all three patients, and regular follow-up was ensured. As a result, clinicians must be aware that serious self-harm can occur during alcohol withdrawal, which can be averted with timely and effective care. Thus, comprehensive education and understanding regarding COVID-19 and its impact on alcohol dependence, its treatment, and recovery are required.

## Data Availability

All the facts and figures underlying the cases are available as part of the article and no additional source data are required.

## Additional Points

*Limitations*. Our study being a case report, we need a larger, multicenter observational study to further analyze alcohol withdrawal and self-harm. It was a single center-based study, and the findings cannot be generalized.
